# Awareness of limited joint mobility in type 2 diabetes in general practice in the Netherlands: an online questionnaire survey

**DOI:** 10.1186/s12875-019-0987-7

**Published:** 2019-07-09

**Authors:** Login Ahmed S. Alabdali, Jasmien Jaeken, Geert-Jan Dinant, Ramon P. G. Ottenheijm

**Affiliations:** 1 Department of Family Medicine, CAPHRI Care and Public Health Research Institute, Maastricht University, PO Box 616, 6200 MD Maastricht, The Netherlands; 2 ICHO, the Centre of Family Medicine, Catholic University, Leuven, Belgium

**Keywords:** Limited joint mobility, Musculoskeletal disorders, Type 2 diabetes mellitus, General practice

## Abstract

**Background:**

Next to the well-known micro- and macrovascular complications, type 2 diabetes mellitus (T2DM) is associated with musculoskeletal disorders of the upper extremities referred to as limited joint mobility (LJM), e.g. carpal tunnel syndrome (CTS) and adhesive capsulitis. Unrecognized and untreated LJM can lead to poor quality of life and non-compliance to diabetes treatment which aggravates LJM. Despite its reported higher prevalence in international prevalence studies, examination of the upper extremities is still no part of the regular diabetes mellitus (DM) check-ups. The primary aim of this study was therefore to evaluate the awareness of Dutch GPs and nurse practitioners concerning LJM. Secondary aims were to evaluate the current management of a patient with LJM, and to assess opinions regarding the question of who should screen for LJM if this is done in the near future.

**Methods:**

An online survey was conducted among 390 general practitioners (GPs) and 245 nurse practitioners (NPs) of three diabetes care groups in The Netherlands to assess their awareness of the association between DM and LJM.

**Results:**

Most GPs are not aware that LJM is a DM complication, with an unawareness for specific upper extremity disorders ranging from 59 to 73%. Of the NPs, 76% is not aware either. Only 41% of GPs would advise the most optimal treatment for diabetes patient with CTS. Finally, only 25% of the GPs believe that screening for LJM should be performed during the regular diabetes check-up compared to 63% of the NPs.

**Conclusion:**

The majority of GPs and NPs are not aware of LJM as a T2DM complication. In contrast to NPs, most GPs do not believe that screening for LJM should be performed during the regular diabetes check-up.

**Electronic supplementary material:**

The online version of this article (10.1186/s12875-019-0987-7) contains supplementary material, which is available to authorized users.

## Background

Diabetes mellitus (DM) is a worldwide healthcare problem with a constantly increasing incidence and prevalence [1]. It is expected that the prevalence will increase by 73% in developing countries and by 20% in developed countries between 2010 and 2030 [2]. In the Netherlands, with a population of approximately 17 million people, more than one million people have been diagnosed with diabetes, and it is estimated that this prevalence will increase by approximately 30% by 2030 [3, 4].

Along with the well-known micro- and macrovascular complications, DM has been associated with musculoskeletal disorders of the upper extremities, referred to as limited joint mobility (LJM). There is no clear definition for LJM. Some authors use the term as a synonym for stiff hand syndrome (cheiroarthropathy), whilst others use it as an umbrella term for a variety of disorders [5–12]. In this article, LJM is used as an umbrella term for specific musculoskeletal disorders of the upper extremities. The most commonly observed specific LJM disorders are trigger finger, Dupuytren’s contracture, carpal tunnel syndrome (CTS) and adhesive capsulitis [13–23]. The prevalence of these disorders is lower in unselected populations: for adhesive capsulitis, 2–5% compared to 5–30% in patients with DM; for trigger finger, 1–2% compared to 5–15%; Dupuytren’s contracture 13% vs 20–63%; and CTS 3.8% vs 25% [24–28].

In the Netherlands, type 2 diabetes mellitus (T2DM) is mainly managed in primary care, where care groups have been established to provide this care. Care groups consist of groups of associated care providers, often exclusively general practitioners (GPs), who are responsible for coordinating and ensuring the delivery of care for patients with T2DM [29, 30]. In practice, GPs delegate most diabetes care activities to nurse practitioners (NPs), who are often employed by these care groups [29]. This diabetes care is delivered in conformity with the Dutch Diabetes Federation Health Care and guidelines of the Dutch College of General Practitioners (DM guidelines) [31, 32]. During periodic diabetes check-ups, GPs and NPs together closely monitor DM patients for blood glucose levels and related complications, e.g. cardiovascular disorders, (poly)neuropathy, retinopathy, and nephropathy. Interestingly, despite the high prevalence of LJM in international studies, evaluation of LJM is still not part of national or international diabetes guidelines, e.g. British and Dutch guidelines, and therefore not incorporated in the check-ups in general practice [33, 34].

LJM can lead to impairments and an inability to perform activities of daily living, but might also negatively affect diabetes treatment. Unrecognized and untreated LJM will lead to a more inactive lifestyle, poorer self-control, earlier appearance of other complications and a decline in the quality of life. In turn, this can aggravate LJM, leading to a vicious circle [26, 31, 35]. So it seems that attention needs to be paid to LJM. We believe that LJM in T2DM patients is underestimated by GPs and NPs. The primary aim of this study was therefore to evaluate the awareness of Dutch GPs and NPs concerning LJM. Secondary aims were to evaluate the current management of a patient with LJM, and to assess opinions regarding the question of who should screen for LJM if this is done in the near future.

## Methods

A cross-sectional descriptive survey to assess GPs’ and NPs’ awareness of common DM complications and co-morbidities, awareness of LJM as a DM complication, management in daily practice, and opinions about screening for LJM as a complication of T2DM was conducted between December 2017 and February 2018. To minimize the burden on GPs and NPs and to optimize the response, a 5–10 min online questionnaire was developed by our research team using Qualtrics software, an online survey tool [36]. It started with an information letter and an online informed consent procedure. The sections of the survey were developed to assess awareness of DM complications and co-morbidities, awareness of LJM as a complication of DM, management decisions in daily practice and opinions about screening. The questions on awareness of DM complications and co-morbidities were based on the DM guidelines, and for those questions only one answer was possible [34]. Questions on awareness of LJM as a DM complication were based on experiences in daily practice and DM guidelines for hand and wrist complaints, in which asking patients about their symptoms and performing a physical examination of the hand and shoulder were considered as screening [37, 38]. Due to the fact that GPs and NPs conduct different tasks, the survey for NPs was varied slightly; GPs see the patients after they have been seen by the NP, and partly respond to the findings of the NP. The questionnaire was informally pilot tested among a small group of NPs, GPs and researchers at our research department (*n* = 10) to evaluate its clarity, clinical relevance, and time required for completion. The questionnaire was developed in a way that it was not possible to look ahead or adjust previous answers.

The questionnaire comprised four sections (see Additional file 1):

1. *Participants’ demographic data:* GPs and NPs were asked about their gender and number of years of experience in practice, while GPs were additionally asked about the type of general practice, number of GPs in practice, GP with special interest (GPwSI), and practice location. NPs were asked about the number of practices where they work.
2. *Medical vignette-based questions about awareness of DM complications and co-morbidities, and their management:* we presented a T2DM patient during his annual check-up with unilateral hand complaints (tingling fingers). GPs were first asked to select the common DM complications they normally screen for during the annual check-up. Second, they were asked to select their initial management for the finger complaints, e.g. expectant policy, make a new appointment, short advice. After these two questions, the vignette continued with the statement that the patient visits again for the finger complaints, and after history taking and physical examination, the diagnosis of CTS is made. Next, GPs were asked about the first treatment step and the follow-up step if the first treatment step was not effective. Finally, GPs were asked if they associated CTS with DM. For NPs we presented the same patient and asked them first to select the diabetes complications (e.g. retinopathy, shoulder complaints) and which physical examination they routinely perform. Next, it was stated that this patient reported tingling in the fingers of one hand, causing limitations in activities of daily life (same case as for GPs). NPs were asked about their policy for this complaint, e.g. expectative, make appointment with GP.
3. *Questions about awareness of common DM complications and co-morbidities, and LJM as a DM complication:* for GPs, 11 disorders were randomly presented, including DM-related complications and co-morbidities (e.g. depression), a disorder that does not have a clear association with DM (osteoporosis), and several upper extremity disorders. This list of disorders was based on the DM guidelines, hand and wrist complaints, and shoulder complaints [34, 37, 38]. On a scale from 1 (DM is certainly NOT a risk factor) to 5 (DM is certainly a risk factor), GPs had to indicate whether DM is a risk factor for developing these disorders. Subsequently, they were asked whether they were familiar with the term “cheiroarthropathy”, and if this was answered positively, they were asked if they could explain this in their own words. Next, three propositions were presented to the GPs with which they could agree or disagree, or indicate that they did not know the answer. NPs received three different propositions addressing questions about DM being a risk factor for musculoskeletal disorders, and upper extremity disorders in particular.
4. *Opinions about screening:* finally, all participants were informed that patients with DM have a higher risk of developing LJM. In view of this risk, they were asked whether screening for LJM should be performed, and if this was answered positively, who should perform this screening: the GP, NP, or someone else.

The survey was approved by the Medical Ethical Assessment Committee of Zuyderland and Zuyd University (METC-Z, ID number: 17-N-165).

### Survey distribution

The survey was conducted in the middle and southern part of the province of Limburg, The Netherlands, where diabetes care is provided by three care groups (Meditta, Zorg In Ontwikkeling (ZIO) and Huisartsen Oostelijk Zuid-Limburg (Huisartsen-OZL)). An email with a link to the online questionnaire was sent to the medical directors of the three care groups, who forwarded this email with link to their affiliated 390 GPs and 245 NPs. After two and four weeks, reminder emails were sent. A brief outline of this survey was contained in the email: participants were informed that the care groups wanted to know if diabetes care can be improved. For that reason, DM-related risk factors were the subject of the questionnaire, but without mentioning that the focus was on LJM.

### Data-analysis

Descriptive analyses were used to summarize the participants’ characteristics and the questionnaire answers. For data analysis, the questionnaire was categorized into four parts: awareness of common DM complications and of LJM, management decisions in daily practice, and opinions on screening (Table 1). To summarize the level of awareness, we dichotomized GPs’ answers by combining the responses ‘I think it is a risk factor’ and ‘certainly risk factor’ into one group (awareness), and the other responses into another group (unawareness).

**Table 1:** Summary of the questions of GPs’ and NPs’ Survey

GP survey (with question number)	
(1) Awareness of DM complications and co-morbidities	
Identifying DM-related complications that should be evaluated during regular DM check-ups (Q1)	
Identifying DM-related complications and co-morbidities according to DM guidelines (Q6)	
Acquainted with the term ‘cheiroarthropathy’ (Q7)	
Acquainted with CTS treatment (Q9)	
(2) Awareness of LJM as a DM complication	
Acquainted with LJM in DM guidelines (Q8)	
Awareness of association between CTS and DM (Q5)	
Awareness of upper extremity complaints in patients with DM (Q6)	
Awareness about relation between hand and wrist complaints in DM patients and their treatment (Q11 + Q12)	
(3) Management decisions in daily practice	
Management of patient with hand/finger complaints during DM evaluation moment (Q2)	
Management of CTS in vignette (Q3 + Q4)	
Management of CTS in daily practice if no corticosteroid injection is given (Q10)	
(4) Opinions on screening	
Should we screen patients with DM for LJM as one DM complication (Q13)	
Who should perform this screening (Q14)	
NP survey (with question number)	
(1) Awareness of DM complications and co-morbidities	
Identifying DM-related complications and co-morbidities that should be evaluated during regular DM check-ups (Q1)	
Identifying physical examination that should be performed during regular DM check-ups (Q2)	
Acquainted with cheiroarthropathy (Q4)	
(2) Awareness of LJM as a DM complication	
Awareness of upper extremity complaints in DM patients (Q5)	
Awareness of hand, wrist, and shoulder complaints in DM patients (Q6)	
Awareness about troubles a DM patient with LJM could experience concerning DM treatment (Q7 + Q8)	
(3) Management decisions in daily practice	
Management of patient with hand/finger complaints during DM evaluation moment (Q3)	
(4) Opinions on screening	
Should we screen patients with DM for LJM as one DM complication (Q9)	
Who should perform this screening (Q10)	

Within the GP group, a subgroup analysis using a chi-square test was performed to assess whether clinical experience or having a special interest (GPwSI) was associated with the outcomes. GPs with > 10 years of experience were considered to be experienced. SPSS software was used (version 25).

## Results

In total, 103 out of 390 emailed GPs (26%) and 122 out of 245 NPs (50%) completed the survey. The participants’ characteristics are presented in Table 2. There were more male (56%; 58/103) than female GP responders (44%; 45/103). The majority of responders were GP principals (99%). Most GPs had > 10 years of clinical experience in general practice (76%; 78/103) and worked in a group practice (39%; 40/103). Some (12%; 12) of the GPs had a special interest, but not one specialized in diabetes. Among NPs, there were more female responders (95%; 116/122) than male responders. The majority of NPs had ≤10 years of clinical experience in general practice (62%; 76/122), and they were employed in one GP practice (66%, 80/122).

**Table 2:** Participants’ demographic data

Demographic data	GP^1^ Total *n* = 103 n (%)	NP^2^ Total *n* = 122 n (%)
Gender		
Male	58 (56)	–
Female	–	116(95)
GP^1^ type		NA
GP principal	102 (99)	
Salaried GP	1 (1)	
Locum GP	0 (0)	
Years of clinical experience in primary care		
< 5	5 (5)	23 (18)
5–10	20 (19)	53 (44)
11–20	40 (39)	44 (36)
> 20	38 (37)	2 (2)
GPwSI^3^	12 (12)	NA
Diabetes	0 (0)	
Cardiovascular	1 (1)	
Musculoskeletal	2 (2)	
Other	9 (9)	
Number of GPs in practice		NA
Single-handed	30 (29)	
Duo-practice	32 (31)	
Group practice	40 (39)	
Other	1 (1)	
Location of practice		NA
Rural	54 (53)	
Urban	48 (47)	
Number of practices worked in	NA	
1		80 (66)
2		32 (26)
> 3		10 (8)

*GP*^*1*^ General practitioner

*NP*^*2*^ Nurse practitioner

*GPwSI*^*3*^ General practitioner with special interest

### Awareness of common DM complications and co-morbidities

In Table 3 we present the results of the common complications. Almost all GPs were aware of the common diabetes complications and co-morbidities. When asked to identify the complications, 78 to 99% correctly identified them, e.g. cardiovascular disorders, retinopathy, depression. In addition, between 93 to 97% indicated that they would evaluate patients for these complications during annual check-ups. About one-third (36%, 37/103) of the GPs answered that they also evaluate for other complications, e.g. sexual disorders. Almost three-quarters of the GPs (72%, 73/102) were aware that osteoporosis is not a DM complication.

**Table 3:** Summary of GPs’ and NPs’ answers for questions about awareness of DM complications & co-morbidities and awareness of LJM as a DM complication, management in daily practice and opinion on LJM screening

	GPs	NPs
(1) Awareness of DM complications and co-morbidities:		
Identifying DM-related complications that should be evaluated during regular DM check-ups.	96–97%*	94–99%*
Identifying DM-related complications and co-morbidities according to DCGP guidelines	78–99%*	NA
Identifying physical examination that should be performed during regular DM check-ups.	NA	96%
Acquainted with the term ‘cheiroarthropathy’ (whether participant can or cannot describe it in their own words).	45%	44.%
Acquainted with CTS treatment.	83%	NA
(2) Awareness of LJM as a DM complication		
Acquainted with LJM in DM guidelines (Q8)	7%	NA
Awareness of association between CTS and DM	24%	NA
Awareness of upper extremity complaints in DM patients	27–41%*	59%
Awareness of hand, wrist, and shoulder complaints in DM patients	NA	23%
Awareness about relation between hand and wrist complaints in DM patients and their treatment compliance.	75%	NA
Awareness about troubles a DM patient with LJM could experience concerning DM treatment	NA	67%
(3) Management decisions in daily practice		
Management of patient with hand/finger complaints during DM evaluation moment	μ	57%
Management of CTS in vignette; corticosteroid injection as a first & second step	41% & 34%	NA
Management of CTS in daily practice if no corticosteroid injection is given	Ϯ	NA
(4) Opinions on screening		
Should we screen patients with DM for LJM as one DM complication (answered with yes)	25%	63%
Who should perform this screening (answered with NPs)	64%	32%

*DM* diabetes mellitus, *DCGP* Dutch College of General Practitioners, *LJM* limited joint mobility, *CTS* carpal tunnel syndrome

**Fig. 1:**
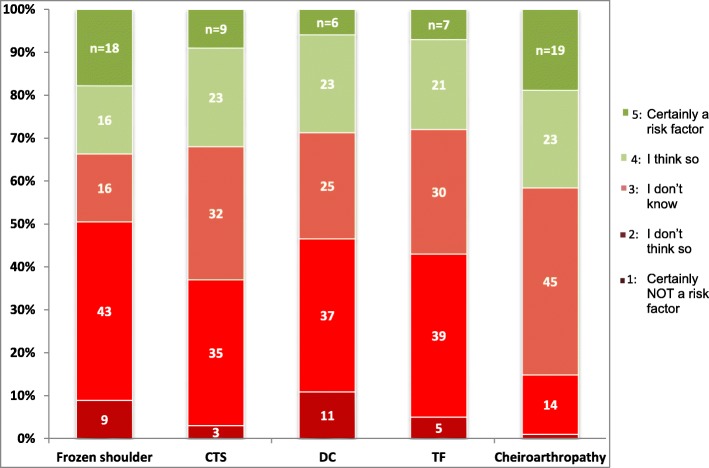
Results of GPs’ awareness questions regarding specific disorders of LJM related to DM. CT: carpal tunnel syndrome; DC: Dupuytren’s contracture; TF: trigger finger. Squares in red are GPs we consider as being unaware

*For multiple answers, the range of the answers was presented; please see Fig. 1 for percentages of each disorder

μ Multiple answers were possible for this question

Ϯ Only answered by 10 GPs; e.g. physiotherapy

When NPs were asked to indicate the complications that should be evaluated during the annual check-up, between 94 and 99% correctly indicated the complications.

Only 10% of GPs (10/101) mistakenly believed that a corticosteroid injection is contraindicated in diabetes patients with CTS, a shoulder disorder or trigger finger, while the majority (83%, 84/101) rightly knew that it is not contraindicated, and 7% (7/101) did not know the answer.

### Awareness of LJM as a DM complication

In Table 3 we present the results of the awareness of LJM in general, while in Fig. 1 the results of the awareness of the specific LJM disorders are pictured. A total of 48% (49/101) of the GPs mistakenly thought that musculoskeletal disorders are included in the DM guidelines as a complication [34], while only 7% (7/101) of the GPs rightly knew that this is not the case, and 45% (45/101) of the GPs did not know the answer. Furthermore, two- thirds of the GPs are unaware of the association between DM and adhesive capsulitis (67%; 68/102) and CTS (69%, 70/102). Additionally, 72% (73/102) of the GPs was unaware of the association between DM and Dupuytren’s contracture, and 73% (74/102) for trigger finger, while 59% (60/102) was unaware of cheiroarthropathy (stiff hand syndrome) being a DM complication. Three-quarters of the GPs (76/101) believed that diabetes patients with hand complaints can experience treatment troubles, while 6% (6/101) did not see this association, and 19% (19/101) did not know the answer.

Fifteen percent of GPs (15/102) was familiar with the term ‘cheiroarthropathy’ and able to explain this term in their own words, while 30% (31/102) of GPs was familiar with this term but was unable to explain it. The rest of the GPs (55%; 56/102) had never heard of it. Less than half (41%; 42/103) of the GPs answered ‘I don’t know’ to the question of whether they think that CTS complaints of the diabetes patient in the medical vignette are associated with DM, while 35% (36/103) answered ‘not related’, and only 24% (25/103) rightly indicated that it is associated. More inexperienced GPs (83% vs. 61%, *p* = 0.047) were unaware that adhesive capsulitis is a DM complication. Additionally, all GPwSI (100% vs. 64%, p 0.044) were unaware that CTS is associated with DM.

Twenty percent of NPs (24/122) answered that they evaluate for hand and wrist disorders, while only 7% (9/122) evaluate for shoulder disorders. About 24% (29/122) of NPs answered ‘agree’ to the question that diabetes patients have complaints of the hand, wrist and shoulder more often than non-diabetes patients, while 24% (29/122) answered ‘I don’t agree’ and 52% (64/122) answered ‘I don’t know’. Twenty-two NPs (18%; 22/122) were familiar with the term ‘cheiroarthropathy’ and were able to explain this term in their own words; 26% (32/122) was also familiar with this term but unable to explain it, and the rest had never heard of it.

### Management

Table 3 presents the results of the management questions. A total of 41% (42/103) of the GPs answered that a corticosteroid injection would be their first treatment step if the diabetes patient was diagnosed with CTS, 19% (20/103) recommended a wait-and-see approach, 17% (18/103) recommended splinting, 13% (13/103) chose to refer to a hospital specialist, while 5% (5/103) recommended analgesics or physiotherapy. If the first treatment step was not effective, 53% (48/90) of the GPs indicated that their next step would be to refer the patient to a hospital specialist, 34% (31/90) recommended a corticosteroid injection, 7% (7/90) chose splinting, 2% (2/90) recommended physiotherapy, and 1% (1/90) advised a wait-and-see policy or analgesics. A minority of GPs thought that corticosteroid injections are contraindicated in DM patients; however, inexperienced GPs are less aware than experienced GPs (21% vs. 6%, p 0.046).

When NPs were asked about their policy when the patient reports unilateral tingling of the fingers causing limitations in activities of daily life, 94% (115/122) answered that they will make an appointment with the GP. Some NPs wrote down in the open field option of this question that they would look for the prayer sign, which is not part of their routine screening procedure.

### Opinions about screening

Both GPs and NPs were finally informed that patients with DM have a higher risk of LJM. When asked if they thought screening for LJM should be performed and, if so, who should perform this screening, one-quarter (25%; 25/99) of the GPs and 63% (77/122) of the NPs felt screening for LJM should be performed during the regular diabetes check-up (Table 3).

When GPs and NPs were invited to provide comments concerning this survey, several GPs (14%; 14/99) made comments, e.g. DM patients are already screened for too many complications, or it’s the patient’s own responsibility to make an appointment with the GP in case of upper extremity complaints. Other GPs believed that screening for LJM would be feasible if performed in a selected group of patients. On the other hand, some NPs (8%; 10/122) responded that they would like to be educated on this topic, that the survey was an eye-opener and that they believe screening for LJM would be a good addition to the regular diabetes follow-up.

## Discussion

The primary aim of this study was to evaluate the awareness of Dutch GPs and NPs concerning LJM. The majority of GPs and NPs demonstrated that they are aware of the common DM complications and co-morbidities, and check for them in practice during the regular DM check-up. However, the majority of GPs were unaware that LJM is also a DM-related complication, with percentages for the various specific disorders ranging from 24 to 41% unaware. The majority of NPs were also unaware that upper extremity disorders are related to DM. About half of the GPs claimed that DM guidelines mention musculoskeletal disorders as a complication (although it is not mentioned), but remarkably, most GPs do not associate DM with adhesive capsulitis, trigger finger, Dupuytren’s contracture, and CTS.

In the medical vignette we presented a 70-year-old DM patient visiting for his annual diabetes check-up. No GPs check for upper extremity disorders, while a minority of the NPs evaluate for hand and wrist disorders (20%) and shoulder disorders (7%). The reason for this might be that GPs associate only feet disorders with DM, or that NPs have a more practical approach to disabilities they observe in patients, e.g. inability to perform blood glucose self-testing. Another reason might be that none of the GPs had a special interest in diabetes. It is also possible that GPs do not screen for it because they have no knowledge about it while the NPs examine symptoms that they commonly see.

In line with the DM guidelines [34], most participants evaluated and examined patients correctly for complications during the DM check-up. Interestingly, despite the fact that the DM guidelines do not mention upper extremity disorders as a complication, DM is mentioned as an important risk factor in the Dutch College of General Practitioners guidelines for ‘hand and wrist complaints’ and ‘shoulder complaints’ [37, 38]. This indicates a discrepancy between the guidelines*.*

For CTS, according to the guidelines for hand and wrist complaints, a corticosteroid injection is recommended as the first treatment step, which can be repeated after two to three weeks if complaints persist [37, 39]. However, only 41% of the GPs recommended an injection as a first treatment step, and if complaints persisted, only 34% would repeat an injection. A corticosteroid injection has been proven to be the most effective treatment option for CTS, since recovery rates are 2.5 times higher compared with placebo [40]. Although only a minority of the GPs thought that a corticosteroid injection is contraindicated, in the medical vignette less than half provided the correct answer. This could be because of a discrepancy between the two guidelines (DM guidelines and hand and wrist complaint guidelines), which might be confusing them.

We observed that experienced GPs are more aware of the association between adhesive capsulitis and DM, and in the medical vignette rightly recommended a corticosteroid injection as the first treatment option for CTS. This may be explained by the fact that over the years, experienced GPs observed this association in clinical practice, and noticed that corticosteroid injections rarely lead to disruption of glucose levels.

In contrast to the GPs, the majority of NPs thought that screening for LJM should be incorporated in the annual check-ups. This difference might be explained by the fact that GPs have less time for a patient compared with the NP. Another explanation could be that GPs question the prevalence and severity of LJM compared to other harmful complications like cardiovascular disorders and nephropathy. Also, they may believe the prevalence of LJM is rather low, so that screening for it would not be cost-effective.

### Strengths and limitations

This is the first study to evaluate the awareness of LJM among Dutch GPs and NPs. Clinical vignettes have been shown to be a valid method to measure clinical practice [41–46]. On most questions a fixed set of answers was given to minimalize differences in clinical interpretation. The survey was developed using the DM guidelines in such a way that there was only one optimal answer. According to other studies [47, 48], response rates are influenced by the length of the survey. Our survey was relatively short, and we observed that indeed most participants did not need more than five minutes to complete it.

The low response rate among the GPs could mean that our findings are influenced by a non-response bias. We tried to overcome non-response bias by sending reminder emails and by mentioning the survey in the periodic newsletter of the care groups. Previous studies have reported a higher response rate using a postal survey method [47]. Concerning the time available for this study, an online approach was most feasible. Other studies using an online survey method among medical professionals achieved higher response rates after using personalized recruitment methods [49, 50]. Unfortunately, to prevent information bias, this personalized approach was not possible in our study, since the primary investigator of the study (RO) is a GPwSI in musculoskeletal disorders and is well known in the region, so this might have affected the answers of GPs and NPs. It is also possible that many GPs and NPs are too busy to read the newsletter or email and may not have been aware of the ongoing survey. Furthermore, the distribution of the survey was dependent on a third party. However, almost all selected answers show a similar trend, indicating that the results from this survey could be representative of a larger group of GPs. Despite the fact that clinical vignettes are a good method to measure practice, differences in interpretation of the questions can be possible. We constructed the vignette and accompanying questions to be as straightforward as possible. We also opted to formulate closed questions for analysis purposes. It is possible that the participants selected the option ‘I don’t know’ although they would have liked to have provided a further explanation. They were given a fixed set of answers for most questions, and it is possible that none of the options in fact corresponded to their opinion. However, we received no comments addressing this topic in the final open question, when GPs and NPs were invited to provide comments regarding this survey. No comments were made about this in the pilot phase either. We noticed that not all of the questions were answered by all participants; it is possible that participants skipped some questions because they were unaware of the right answer.

#### Implications for future research

The decision of whether to incorporate screening for LJM in the annual check-ups of diabetes patients depends among other things on the prevalence of LJM. Future studies are needed to investigate the prevalence of LJM in the Dutch population. To prevent unnecessary screening, it is possible that a certain subgroup of diabetes patients is more at risk of developing LJM, e.g. subgroups selected on the duration of disease, and screening could be confined to them. Further research is advised to evaluate the GPs’ reasons for not being willing to screen for LJM.

#### Implications for practice

Awareness among GPs and NPs about the fact that LJM is a DM-related complication should be improved. This seems a task for guideline committees and providers of continuing medical education; we do not encourage incorporating LJM screening in the DM guidelines yet, before LJM prevalence studies are done. When it is decided to incorporate it in the annual check-ups, the question will arise of who should perform this screening. Our study showed that in contrast to GPs, NPs are willing to perform this task. Currently, NPs are not trained for this. Appropriate training of NPs is a prerequisite in their education when deciding to delegate screening for LJM to these professionals.

## Conclusion

The results of this survey among GPs and NPs show that, despite excellent knowledge of common complications, the majority is unaware that LJM is also a DM-related complication. The majority of GPs think that screening for LJM should not be their task, while NPs are not trained for LJM screening.

## Additional file

Additional file 1: Online survey of general practitioners and nurse practitioners (translated from Dutch). (DOCX 24 kb)

## Data Availability

The dataset of this study is available from the corresponding author on reasonable request.

## Abbreviations

CTS: Carpal Tunnel Syndrome
DM: Diabetes Mellitus
GP: General practitioner
GPwSI: General practitioner with special interest
LJM: Limited joint mobility
NP: Nurse practitioner
T2DM: Type 2 diabetes mellitus
