# Supplementary material for: Awareness of limited joint mobility in type 2 diabetes in general practice in the Netherlands: an online questionnaire survey
Source: BMC Fam Pract. 2019 Jul 9;20:98. doi: 10.1186/s12875-019-0987-7 (PMC6615429; doi:10.1186/s12875-019-0987-7)
Supplement: Supplementary file 1 — Online survey of general practitioners and nurse practitioners (translated from Dutch). (DOCX 24 kb) [file 12875_2019_987_MOESM1_ESM.docx]

**Supplementary Data**

**Online survey of general practitioners (translated from Dutch)**

**Part 1: Demographic data**

**Gender**

1. Male
2. Female

**GP type**

1. GP principal
2. Salaried GP
3. Locum GP

**Type of GP practice**

1. Solo-practice
2. Duo-practice
3. Group practice
4. I am a salaried GP
5. I am a locum GP

**GP with special interest**

1. No
2. Yes, diabetes mellitus
3. Yes, cardiovascular disease
4. Yes, musculoskeletal system
5. Yes, other

**Duration (in years) since qualification as GP**

1. <5
2. 5 – 10
3. 11 – 20
4. >20

**Practice location**

1. Urban
2. Rural

**Part 2: Medical vignette**

A 70-year-old male with diabetes mellitus type 2 (non-insulin dependent) visits you for his annual diabetes check-up.

**Q1. During the annual check-up, you evaluate several diabetes-related complications. Which of the following apply? Multiple answers possible.**

1. Neuropathy
2. Cardiovascular risk factors
3. Nephropathy
4. Retinopathy
5. Other:

**Q2. The patient tells you that since 4 weeks, he has a tingling feeling in all fingers of his right hand, and that he cannot open properly a bottle cap. The complaints cause an obstruction of daily functioning. He has no neurological feet symptoms. What do you do during THIS CONSULTION? Multiple answers possible.**

1. Expectative
2. You invite the patient to make a new appointment
3. You provide a short advice
4. You refer the patient to the nurse practitioner
5. Other:

**Q3. This patient visits you again. After history taking and physical examination, you establish the diagnosis ‘carpal tunnel syndrome’ (CTS). What is your FIRST treatment step?**

1. Expectative
2. A splint
3. Analgesics
4. Corticosteroid injection
5. Physiotherapy
6. Referral to hospital (for example a neurologist) (*If this option is selected, the next question is skipped).*

**Q4. A few weeks later, the patient visits you again with unchanged symptoms. Your previous treatment was not effective. What is your NEXT treatment step?**

1. Expectative
2. A splint
3. Analgesics
4. Corticosteroid injection
5. Physiotherapy
6. Referral to hospital (for example a neurologist)

**Q5. Do you think the patient’s CTS is related to his diabetes mellitus?**

1. Yes
2. No
3. I do not know

**Part 3: Risk factors and propositions**

**Q6. For the following conditions indicate, on a scale from 1 to 5, whether diabetes mellitus is a risk factor (1= diabetes is certainly NOT a risk factor, 5= diabetes is certainly a risk factor).**

|  | **1: diabetes is certainly NOT a risk factor** | **2: I don’t think so** | **3: I don’t know** | **4: I think so** | **5: diabetes is certainly a risk factor** |
| --- | --- | --- | --- | --- | --- |
| **Cardiovascular diseases** |  |  |  |  |  |
| **Frozen shoulder** |  |  |  |  |  |
| **Depression** |  |  |  |  |  |
| **Neuropathy** |  |  |  |  |  |
| **De Quervain tenosynovitis** |  |  |  |  |  |
| **CTS** |  |  |  |  |  |
| **Schizophrenia** |  |  |  |  |  |
| **Osteoporosis** |  |  |  |  |  |
| **Hand osteoarthritis** |  |  |  |  |  |
| **Dupuytren’s contracture** |  |  |  |  |  |
| **Foot ulcer** |  |  |  |  |  |
| **Trigger finger** |  |  |  |  |  |
| **Cheiroarthropathy (stiff hand syndrome)** |  |  |  |  |  |

**Q7. I am familiar with the term ‘cheiroarthropathy’ *(stiff hand syndrome)***

1. I know what this term means (I can explain it in my own words)
2. I have heard of it, but I cannot explain this in my own words
3. Never heard of it

**There are now a number of short** propositions**. You answer these statements with 'Agree’, Disagree' or 'I do not know**'.

|  | **Agree** | **Disagree** | **I don’t know** |
| --- | --- | --- | --- |
| **Q8**. According to the current Dutch GP guidelines ‘Diabetes Mellitus type 2’, diabetes patients have an increased risk of musculoskeletal complaints. |  |  |  |
| **Q9**. Diabetes mellitus is a contra-indication for administering a corticosteroid injection in the treatment for CTS, shoulder complaints, trigger finger.  **Q10**. If option ‘Agree’ is selected: Explain your answer. (free text) |  |  |  |
| **Q11**. A diabetes patient with hand and wrist complaints can experience problems concerning his/her diabetes treatment.  **Q12**. If option ‘Agree’ is selected: How do you explain this? (free text) |  |  |  |
| Research has shown that diabetes patients have an increased risk of developing musculoskeletal complaints like cheiroarthropathy, trigger finger, Dupuytren’s contracture, frozen shoulder and CTS.  **Q13**. Do you find, in view of the above, that diabetes patients have to be screened for musculoskeletal complaints?  **Q14**. If option ‘Agree’ is selected: Who should perform this screening?  The general practitioner  The nurse practitioner  Someone else, namely: (free text) |  |  |  |

**Q15. Do you have any further comments about this study or questionnaire? (free text)**

**Online survey of nurse practitioners (translated from Dutch)**

**Part 1: Demographic data**

**Gender**

1. Male
2. Female

**Duration (in years) qualified as nurse practitioner diabetes**

1. <5
2. 5 – 10
3. 11 – 20
4. >20

**In how many GP practices do you work?**

1. 1
2. 2
3. >2

**Part 2: Medical vignette**

A 70-year-old male with diabetes mellitus type 2 (non-insulin dependent) visits you for his annual diabetes check-up.

**Q1. For which of the following conditions is diabetes mellitus type 2 a risk factor? (Multiple answers are possible).**

1. Retinopathy
2. Neuropathy
3. Cardiovascular diseases
4. Sexual dysfunctions
5. Nephropathy
6. Foot complaints
7. Hand and wrist complaints
8. Shoulder complaints
9. Other: (free text)

**Q2. What physical examination do you perform during this consultation?**

1. Measuring blood pressure
2. Measuring weight
3. Examination of the feet
4. Examination of the hands
5. Other: (free text)

**Q3. The patient tells you that in the past 4 weeks, he has had a tingling feeling in all fingers of his right hand, and that he cannot properly open a bottle cap. The complaints hinder daily functioning. He has no neurological feet symptoms. What do you do during THIS CONSULTION? Multiple answers are possible.**

1. Expectative
2. Analgesics
3. Physiotherapy
4. Make an appointment with the GP
5. Provide information about the importance of therapy adherence to maintaining a stable glucose level
6. Other: (free text)

**Q4. I am familiar with the term ‘cheiroarthropathy’ *(stiff hand syndrome)***

1. I know what this term means (I can explain it in my own words)
2. I have heard of it, but I cannot explain it in my own words
3. Never heard of it.

**Part 3: Risk factors and propositions**

|  | **Agree** | **Disagree** | **I don’t know** |
| --- | --- | --- | --- |
| Q4. Patients with diabetes have complaints of the musculoskeletal system more often than patients without diabetes |  |  |  |
| Q6. Patients with diabetes have complaints of the hand, wrist and shoulder more often than patients without diabetes |  |  |  |
| Q7. A patient with diabetes and hand and wrist complaints can experience some troubles concerning his/her diabetes treatment.  Q8. If agree: explain |  |  |  |

**Q9.** Research has shown that patients with diabetes have an increased risk of developing musculoskeletal complaints like cheiroarthropathy, trigger finger, Dupuytren’s contracture, frozen shoulder and CTS.

In view of the above, do you feel that diabetes patients should be screened for musculoskeletal complaints?

1. Yes
2. No
3. I don’t know

**Q10. If agree: who should perform this screening?**

The general practitioner

The nurse practitioner

Someone else, namely: (free text)

**Q11. Do you have any further comments about this study or questionnaire? (Free text)**
